# Integrating multiple protein-protein interaction networks to prioritize disease genes: a Bayesian regression approach

**DOI:** 10.1186/1471-2105-12-S1-S11

**Published:** 2011-02-15

**Authors:** Wangshu Zhang, Fengzhu Sun, Rui Jiang

**Affiliations:** 1 MOE Key Laboratory of Bioinformatics and Bioinformatics Division, TNLIST/Department of Automation, Tsinghua University, Beijing, 10084, China; 2 Molecular and Computational Biology program, University of Southern California, Los Angeles, CA90089, USA

## Abstract

**Background:**

The identification of genes responsible for human inherited diseases is one of the most challenging tasks in human genetics. Recent studies based on phenotype similarity and gene proximity have demonstrated great success in prioritizing candidate genes for human diseases. However, most of these methods rely on a single protein-protein interaction (PPI) network to calculate similarities between genes, and thus greatly restrict the scope of application of such methods. Meanwhile, independently constructed and maintained PPI networks are usually quite diverse in coverage and quality, making the selection of a suitable PPI network inevitable but difficult.

**Methods:**

We adopt a linear model to explain similarities between disease phenotypes using gene proximities that are quantified by diffusion kernels of one or more PPI networks. We solve this model via a Bayesian approach, and we derive an analytic form for Bayes factor that naturally measures the strength of association between a query disease and a candidate gene and thus can be used as a score to prioritize candidate genes. This method is intrinsically capable of integrating multiple PPI networks.

**Results:**

We show that gene proximities calculated from PPI networks imply phenotype similarities. We demonstrate the effectiveness of the Bayesian regression approach on five PPI networks via large scale leave-one-out cross-validation experiments and summarize the results in terms of the mean rank ratio of known disease genes and the area under the receiver operating characteristic curve (AUC). We further show the capability of our approach in integrating multiple PPI networks.

**Conclusions:**

The Bayesian regression approach can achieve much higher performance than the existing CIPHER approach and the ordinary linear regression method. The integration of multiple PPI networks can greatly improve the scope of application of the proposed method in the inference of disease genes.

## Background

Inference of genes responsible for human inherited diseases has been one of the major tasks in modern human and medical genetics. Traditionally, associations between diseases and genes are pinpointed through statistical methods such as family-based linkage analysis and population-based association studies [1], which have been demonstrating remarkable successes in mapping disease genes. However, linkage analysis can only associate diseases with genetic regions that typically contain dozens to hundreds of genes, and association studies usually require carefully selected candidate genes that are biologically related to the disease of interest, making computational inference of causative genes from positional candidates and the selection of functional candidates indispensible [2,3].

Most existing computational methods for inferring causative genes from candidates are formulated as a one-class novelty learning problem that is usually solved with the guilt-by-association principle, which suggests to compute a score from functional genomics data to quantify the strength of association between a query disease and a candidate gene, and then rank candidate genes according to their scores to facilitate the selection of susceptibility genes [4]. For this purpose, various genomic data, including protein sequences [5,6], gene expression profiles [6-8], functional annotations [6,8-11], literature descriptions [6,7,12], protein interactions [6,8,13,14], and many others [15] have been employed to characterize similarities between genes, with the assumption that genes similar in one or more characteristics are usually similar in their functions, and thus are likely to be associated with the same disease. Recent studies have also shown the modular nature of human genetic diseases [15-23], which suggests that diseases share common clinic characteristics are often caused by functionally related genes [24]. With this understanding, various methods have been proposed to utilize phenotype similarity and gene proximity for the inference of causative genes for human inherited diseases [14,25-27].

It has been shown that the Pearson's correlation coefficient of similarities between phenotypes and closeness of genes in a single protein-protein interaction (PPI) network can be used as a concordance score to facilitate the prioritization of candidate genes [25]. However, PPI networks are far from complete. For example, the Human Protein Reference Database (HPRD) [28], as one of the most comprehensive protein interaction databases, only covers less than half of human protein-coding genes. Therefore, relying on a single PPI network to infer disease genes will restrict the scope of application of such methods. Meanwhile, there have been a few protein interaction databases constructed and maintained independently. These databases are often quite diverse in coverage and quality, making the selection of a suitable PPI network inevitable. Moreover, although the naïve thinking of combining all available protein interactions into a single large network is straightforward, performance of methods based on such a combined network is questionable [25].

With these considerations, we propose a Bayesian regression approach that can be used with either a single PPI network or multiple networks to prioritize candidate genes. We adopt a linear model to explain disease similarity using gene proximity, and we solve this model via a Bayesian approach, which yields an analytic form of Bayes factor for measuring the strength of association between a query disease and a candidate gene. We then use Bayes factors as scores to prioritize candidate genes. We show the validity of assumptions of this approach, and we demonstrate the effectiveness of this approach on five PPI networks via large scale leave-one-out cross-validation experiments and comprehensive statistical analysis. We further show the capability of our approach in integrating multiple PPI networks.

## Methods

### Data sources

We propose to infer disease genes using gene proximity profiles derived from protein-protein interaction (PPI) networks, a phenotype similarity profile calculated using text mining technique, and known associations between disease phenotypes and genes extracted from the Online Mendelian Inheritance in Man (OMIM) database.

There have been a few PPI networks with diverse coverage and quality. In our study, we adopt five widely-used PPI networks to calculate gene proximity profiles. First, the Human Protein Reference Database (HPRD) contains human protein-protein interactions that are manually extracted from the literature by expert biologists [28]. After removing duplications and self-linked interactions, we extract from release 8 of this database 36,634 interactions between 9,470 human genes. Second, the Biological General Repository for Interaction Datasets (BioGRID) contains protein and genetic interactions of major model organism species [29]. We extract from version 2.0.63 of this database 29,558 interactions between 9,043 human genes. Third, the Biomolecular Interaction Network Database (BIND) contains both high-throughput and manually curated interactions between biological molecules [30]. From this database, we collect 14,955 interactions between 6,089 human genes. Fourth, the IntAct molecular interaction database (IntAct) contains protein-protein interaction derived from literature [31]. From this database, we collect 30,030 interactions between 6,775 human genes. Finally, the Molecular INTeraction database (MINT) contains information about physical interactions between proteins [32]. From this database, we collect 15,902 interactions between 7,200 human proteins. Details about these five PPI networks are given in Table 1.

**Table 1:** Summary of the five protein-protein interaction networks.

	Genes	Interactions	Seed diseases	Seed genes	Seed associations
HPRD	9,470	36,634	1,590	1,440	2,466
BioGRID	9,043	29,558	1,412	1,247	2,166
BIND	6,089	14,955	1,016	811	1,442
IntAct	6,775	30,030	1,094	933	1,622
MINT	7,200	15,902	889	677	1,231

The phenotype similarity profile, which is obtained from an earlier work of van Driel *et al*[21], is represented as a matrix of pair-wise similarities between human disease phenotypes. Briefly, van Driel *et al* analyzed the full-text and clinical synopsis fields of all OMIM records, and used the anatomy and the disease sections of the medical subject headings vocabulary (MeSH) to extract terms from the OMIM records [21]. By doing this, they were able to characterize a phenotype using a feature vector that was composed of standardized and weighted phenotypic feature terms and further calculated a similarity score for a pair of phenotypes as the cosine of the angle of their feature vectors. Finally, they obtain a phenotype similarity profile that contains pair-wise similarity scores for 5,080 OMIM diseases.

Known associations between disease phenotypes and genes are extracted from BioMart [33]. For genes in HPRD, we obtain 2,466 associations between 1,590 diseases and 1,440 genes. For BioGRID, we obtain 2,166 associations between 1,412 diseases and 1,247 genes. For BIND, we obtain 1,442 associations between 1,016 diseases and 811 genes. For IntAct, we obtain 1,622 associations between 1,094 diseases and 933 genes. For MINT, we obtain 1,231 associations between 889 diseases and 677 genes. We also summarize the above information in Table 1.

### Bayesian linear regression

We adopt a linear regression model to explain disease similarities in the phenotype similarity profile using gene similarities in one or more gene proximity profiles, and we solve this regression model via a Bayesian approach [34]. For a clear presentation, we first derive this method using a single gene proximity profile and then extend this model to include multiple profiles.

A gene proximity profile contains pair-wise similarity measure of every two genes and is calculated as the diffusion kernel of the underlying PPI network. Given a network of *n* nodes, represented by an adjacency matrix A, we calculate the Laplacian of the network as **L = D – A** and the diffusion kernel as Z = *e^-γ^*^L^, where **D** is a diagonal matrix containing node degrees, and 0 <*γ* < 1 a free parameter that controls the magnitude of diffusion. With the kernel **Z** = (*z_ij_*)*_n×n_*, we define the proximity of two genes *i* and *j* as the corresponding element *z_ij_* in the kernel.

Let *y_dd′_* denote the similarity score between a query disease *d* and another disease *d′*. We define the phenotype similarity vector for disease *d* as , i.e., the similarities between disease *d* and all *m* diseases *d*_1_,*d*_2_,…,*d_m_* in the phenotype similarity profile. Let *Z_gg′_* denote the proximity score between genes *g* and *g′* in the gene proximity profile and *G*(*d*) the set of genes known as associated with disease *d*. We define the proximity between gene *g* and disease *d* as the summation of proximity scores between gene *g* and all genes known as associated with disease *d*, denoted by . We further define the gene proximity vector for gene *g* as , i.e., the proximities between gene *g* and all diseases *d*_1_,*d*_2_,…,*d_m_* in the phenotype similarity profile.

We then explain the phenotype similarity vector for disease *d* using gene proximity vectors of all genes that are associated with the disease via a linear regression model

,

where **y** = **y***_d_* is the response vector, **X** the design matrix, **β** the coefficient vector, and  the residual vector. For disease *d* associated with a total of *p* genes, the design matrix **X** has *p +* 1 columns, with the first column being 1s for the purpose of incorporating the intercept.

We solve this model using a Bayesian approach [34] and use the resulting Bayes factor to measure the strength of evidence for a candidate association. For the alternative model, we assume that **y** conditional on **X** is subject to a normal distribution, as

,

with residuals independent and identically distributed, following normal density with mean 0 and variance *σ*^2^. We set conjugate prior distributions for **β** and *σ*^2^, as

,

where  is composed of prior means, and *σ*^2^Σ prior variances with  being a diagonal matrix. The joint distribution of all random quantities **y**, **β**, and *σ*^2^ is then given as.

Integrating out **β** and *σ*^2^, we obtain the marginal likelihood of **y** given **X** as

,

where  and  with  and .

On the other hand, for the null model, where **y** is independent of **X**, the marginal likelihood of **y** can be derived in a similar way, as

,

where , and .

Then, the Bayes factor is calculated as the ratio of the marginal likelihood under the alternative and the null hypotheses, respectively, as.

Following literature [34], we will use the parameter setting (as +∞ in calculation) and *σ_i_ =* 1 (for *i ≥* 1) throughout this paper, though a grid search for other values of *σ_i_* shows that the method is quite robust to this parameter. It has been shown that the parameter *γ* should take a small value [35-37]. In our study, we perform a grid search for this parameter and find results are quite robust when 0.1 ≤ *γ* ≤ 0.3. Therefore we will use *γ* = 0.2 throughout this paper.

Obviously, a larger Bayes factor indicates a better exhibition of the linear relationship between the disease similarity and the gene proximity. With this understanding, we propose the following schemes to prioritize candidate genes. First, given a query disease and a set of candidate genes, we calculate a Bayes factor for each candidate gene, with the assumption that the gene is the only one associated with the query disease. Then, we rank candidate genes in non-increasing order according to their Bayes factors. This scheme mimics the situation in which we aim at inferring associations between genes and a "novel" disease that has yet not been previously studied. Second, for a disease that has been previously studied (and thus already has some genes associated), we can choose to calculate Bayes factors for candidate genes with the inclusion of the genes that are already known to be associated with the disease. This scheme is more suitable for inferring associations between genes and a disease that has been previously studied (and thus has known associated genes).

In the case that multiple gene proximity profiles calculated from multiple PPI networks are available, we extend the regression model by incorporating additional gene proximity vectors into the design matrix. Suppose that disease *d* is associated with *p* genes, and *q* gene proximity profiles are available, the design matrix **X** will have *pq* + 1 columns, with column 1 for the intercept, columns 2 to *p* + 1 for the first profile, columns *p* + 2 to 2*p* + 1 for the second profile, and so on. With this extension, all the above reasoning remains unchanged.

### Validation methods and evaluation criteria

We adopt two large scale leave-one-out cross-validation experiments to test how well the Bayesian regression approach performs in recovering known associations between diseases and genes. In the validation of random controls, we prioritize genes that are known as associated with diseases against randomly selected genes. In each run of the validation, we select an association between a gene and a disease, assume that the association is unknown, and prioritize the gene against a set of 99 randomly selected control genes. In the validation of simulated linkage intervals, we simulate the real situation of identify disease genes by prioritizing genes that are known as associated with diseases against genes that are located around the disease genes. In each run of the validation, we select an association between a gene and a disease, assume that the association is unknown, and prioritize the gene against a set of control genes that are located in 10Mbp upstream and downstream around this gene. In both experiments, we adopt the first scheme to mimic the situation of inferring associations between genes and novel diseases, for the purpose of achieving a more strict validation.

In each of the above leave-one-out cross-validation experiments, we repeat the validation run for every known association between a disease and a gene, obtaining a number of ranking lists. We further normalize the ranks by dividing them with the total number of candidate genes in the ranking list to obtain rank ratios and derive two criteria to measure the performance of a prioritization method. The first criterion is mean rank ratio, which is simply the average of rank ratios over all disease genes in a cross-validation experiment. This criterion provides a summary of the ranks of all genes that are known as associated with diseases, and the smaller the mean rank ratio, the better a method. The second criterion is AUC, the area under the receiver operating characteristic curve (ROC). Given a list of rank ratios and a predefined threshold, we define the sensitivity as the percentage of disease genes that are ranked above the threshold and the specificity as the percentage of control genes that are ranked below the threshold. Varying the threshold values, we are able to plot a ROC curve, which shows the relationship between sensitivity and 1-specificity. Calculating the area under the ROC curve, we obtain the AUC score, which provides an overall measure for the performance of a prioritization method.

## Results

### Gene proximity implying phenotype similarity

The proposed approach for inferring disease genes is based on the assumption that phenotypically similar diseases are caused by functionally related genes that are usually proximal in a PPI network. Moreover, we assume the existence of a linear relationship between similarities of diseases and proximities of genes that are associated with the diseases. In order to validate this assumption, we compile from HPRD 2,466 associations between 1,590 diseases and 1,440 genes, calculate Bayes factors for these disease genes, and run a Wilcoxon signed rank test to check whether the resulting Bayes factors are significantly greater than 1 (the random case). Results show that the *p*-value is smaller than 2.2E-16, indicating that the similarities of diseases have a linear relationship with the proximities of disease genes.

**Figure 1:**
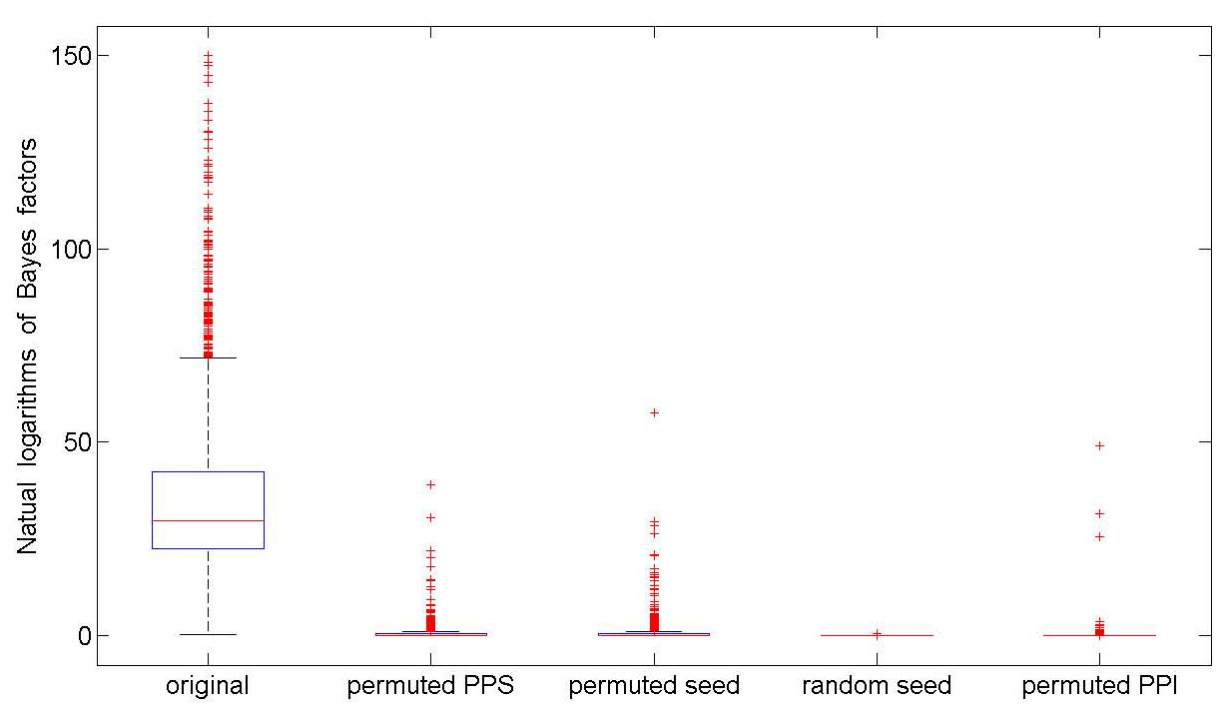
**Bayes factors of the original and permuted data.** “original”, “permuted PPS”, “permuted seed”, “random seed”, “permuted PPI” denote the results obtained using original data, permuting phenotype similarity profile, permuting disease-gene associations, replacing disease genes in disease-gene associations with randomly selected genes, and permuting connections in the protein-protein interaction network, respectively.

To further substantiate this point, we perform a series of permutations towards disease-disease, disease-gene, and gene-gene relationships. First, we break disease-disease relationship by permuting the phenotype similarity profile. Second, we break disease-gene relationship by two methods: (1) permuting disease-gene associations and (2) replacing disease genes in known disease-gene associations with randomly selected genes. Third, we break gene-gene relationship by permuting connections in the underlying protein-protein interaction network while keeping node degrees and recalculating the diffusion kernel. For each of the above permutations, we calculate Bayes factors of disease genes and present the results in Figure 1, from which we can clearly see that the median of Bayes factors based on the original data is much higher than those using permuted relationships.

We also perform similar studies using data extracted from BioGRID, BIND, IntAct, and MINT, and we obtain similar results as HPRD. From these comprehensive studies, we conclude that similarities between diseases can be explained using network proximities of genes that are associated with the diseases. In other words, gene proximity implies phenotype similarity.

### Prioritization with individual PPI networks

**Figure 2:**
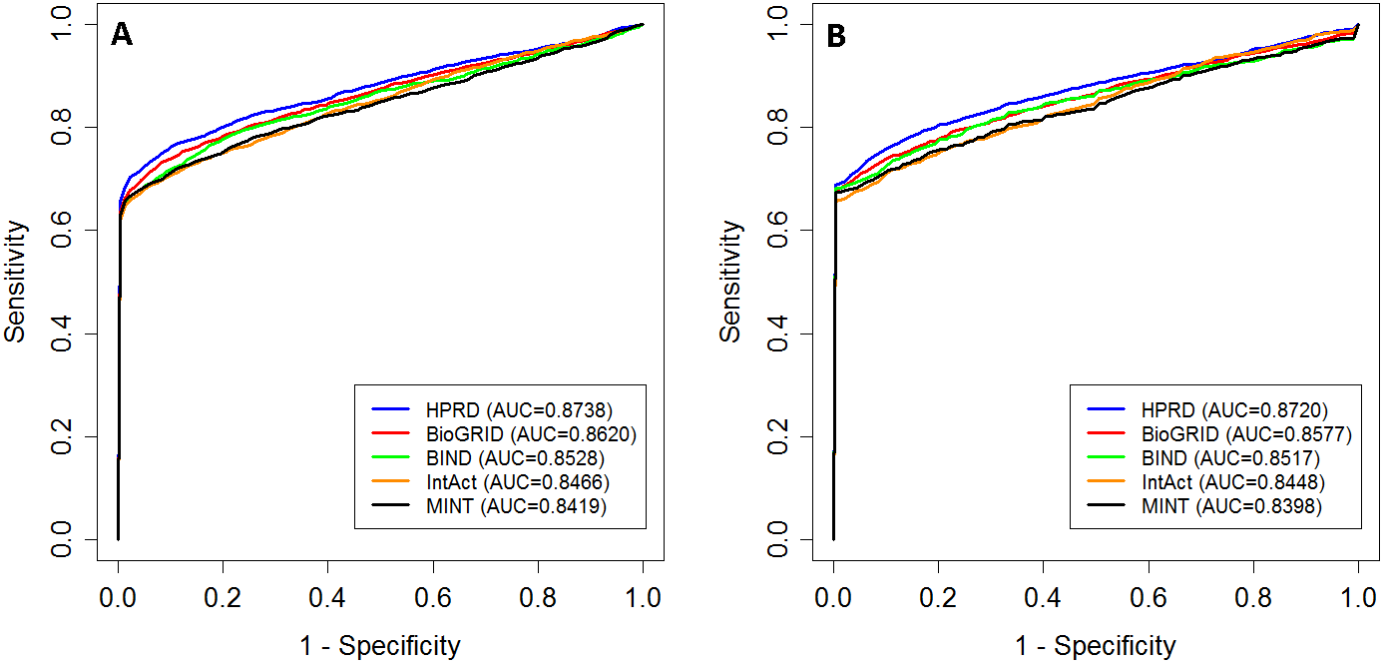
**ROC curves of the five PPI networks on random controls (A) and linkage intervals (B).** AUC scores for the validation of random controls are average of 10 independent runs.

We design a series of large scale leave-one-out cross-validation experiments to show the validity and effectiveness of the Bayesian regression approach on individual PPI networks. As described in the method section, in each run of the validation procedure, we prioritize candidate genes according to the Bayes factors against two control sets, random controls and linkage intervals, with the performance being evaluated by mean rank ratios and AUC scores. Results are shown in Table 2 and Figure 2.

**Table 2:** Performance of the Bayesian regression approach on individual data sources.

	Random Control		Linkage Interval	
	Mean Rank Ratio (SD)	AUC (SD)	Mean Rank Ratio	AUC

HPRD	0.1349 (0.0004)	0.8738 (0.0004)	0.1353	0.8720
BioGRID	0.1466(0.0003)	0.8620 (0.0003)	0.1495	0.8577
BIND	0.1557(0.0007)	0.8528 (0.0008)	0.1556	0.8517
IntAct	0.1618(0.0004)	0.8466 (0.0004)	0.1623	0.8448
MINT	0.1665(0.0006)	0.8419 (0.0006)	0.1674	0.8398

Results are obtained using diffusion kernel (*γ=*0.2) with Bayesian prior *µ=*0 and *σ_i_ =*1 (for *i ≥* 1). Results for the validation of random controls are mean (standard deviation) of 10 independent runs.

From Table 2, we see that the mean rank ratios obtained using the five PPI networks are all below 0.17, and the AUC scores are all above 0.83, suggesting the effectiveness of the Bayesian regression approach. The best performance are obtained using HPRD, with which the mean rank ratios against random controls and linkage intervals are 0.1349 and 0.1353, respectively, and the AUC scores are 0.8738 and 0.8720, respectively. From Figure 2, we see that the ROC curves of the HPRD data set are above those of the other data sets, suggesting that the performance on HPRD is superior over that of the others. To understand this observation, we perform one-sided Wilcoxon rank sum test against the hypothesis that Bayes factors of disease genes for the HPRD data set are greater than those for the other data sets. Results show that Bayes factors of disease genes for the HPRD data set are indeed greater than those of BioGRID (*p*-value=4.7E-2), BIND (*p*-value=2.6E-3), IntAct (*p*-value=1.9E-5), and MINT (*p*-value=2.5E-5). We therefore conjecture that the performance of the proposed method depends on how well the linear relationship between disease similarity and gene proximity exhibits.

**Figure 3:**
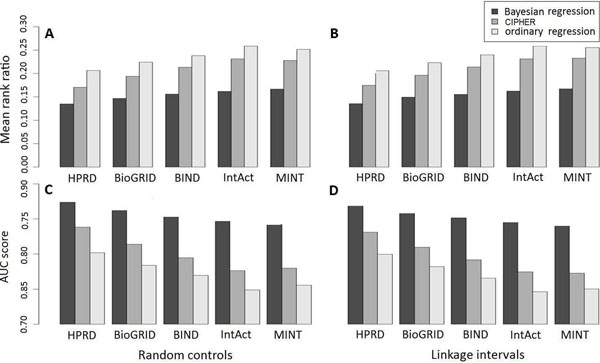
**Comparison with the CIPHER approach and the ordinary regression method.** Subplots A and C illustrate mean rank ratios and AUC scores against random controls, respectively. Subplots B and D illustrate mean rank ratios and AUC scores against linkage intervals, respectively. Results for the validation of random controls are average of 10 independent runs (Variance not shown).

To further demonstrate the effectiveness of the proposed approach, we repeat the same leave-one-out cross-validation experiments using the existing CIPHER approach [25], which relies on Pearson's correlation coefficient between the disease similarity vector and the gene proximity vector to prioritize candidate genes. We compare the results of these two approaches in Figure 3, from which we see clearly that the Bayesian regression approach in general achieves lower mean rank ratios and higher AUC scores in all the five data sets. For example, when performing cross-validation for linkage intervals using the HPRD data set, the CIPHER approach achieves a mean rank ratio of 0.1746 and an AUC score of 0.8313, whereas the Bayesian approach achieves a mean rank ratio of 0.1353 and an AUC score of 0.8720, suggesting an obvious improvement over the CIPHER approach. Note that the CIPHER method calculates gene proximity matrix by applying a Gaussian kernel to the shortest path distance matrix of the underlying network. We also try to use the diffusion kernel matrix as the gene proximity matrix and find the difference is not obvious. These results strongly suggest that the Bayesian regression approach is superior over the CIPHER approach in prioritizing candidate genes.

It is also of interest to compare the Bayesian approach with the ordinary linear regression method. For this purpose, we implement another method that relies on *R*^2^, the coefficient of determination, to prioritize candidate genes. We repeat the leave-one-out cross-validation experiments for this method and present the results in Figure 3, from which we see clearly that the Bayesian regression approach in general achieves higher performance than the ordinary regression method in terms of both mean rank ratios and AUC scores in all the five data sets.

### Prioritization with the integration of multiple PPI networks

The coverage of a single PPI network is in general not high. Even the largest HPRD network covers only 9,470 genes, less than half of known human protein-coding genes. We therefore propose to use the Bayesian regression approach to integrate multiple PPI networks, for the purpose of improving the coverage.

**Figure 4:**
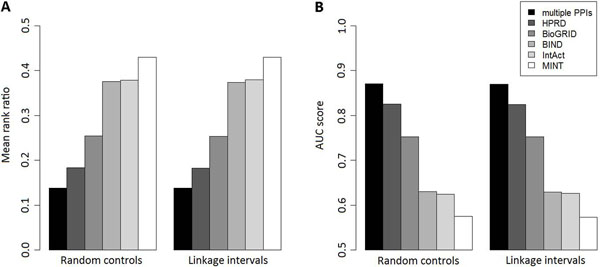
**Performance of the integration method.** Subplot A illustrates mean rank ratios for the integration method and individual PPI networks. Subplot B illustrates AUC scores for the integration method and individual PPI networks. Results for the validation of random controls are average of 10 independent runs (variance not shown).

By taking the union of genes on individual PPI networks, we obtain 15,644 human genes. Focusing on these genes, we extract from BioMart 2,708 associations between 1,752 diseases and 1,621 genes. With this data set, we repeat the leave-one-out cross-validation experiments using individual gene proximity profiles and present the results in Figure 4. Note that in this procedure, we set the proximity of two genes to zero (minimum proximity) if any of the two genes is absent from the underlying network. We observe that the performance on individual proximity profiles drops dramatically in this larger data set (in comparison with Table 2), simply because each PPI network covers only a fraction of genes, and the scheme of handling missing data (setting to zero) yield small Bayes factors for genes that are absent from the network.

We then use the Bayesian regression approach to integrate all the five PPI networks by extending the design matrix to include gene proximities from multiple profiles. We repeat the leave-one-out cross-validation experiments and present the results in Figure 4, from which we observe clearly the better performance of the proposed approach with the integrated use of multiple PPI networks. The mean rank ratios for random controls and linkage intervals are 0.1385 and 0.1380, respectively, with AUC scores being 0.8702 and 0.8692, respectively. In contrast, combining all genes and interactions in individual PPI networks together to form a large network (15,644 nodes and 77,332 edges) and then applying CIPHER only yields mean rank ratios 0.1850 and 0.1876 (AUC scores 0.8230 and 0.8180) for random controls and linkage intervals, respectively. Directly applying the Bayesian regression approach to the combined network yields mean rank ratios 0.1462 and 0.1469 (AUC scores 0.8624 and 0.8601) for random controls and linkage intervals, respectively. Furthermore, we also extract from the combined network interactions that exist in at least two individual PPI networks and obtain a high confident network (8,463 nodes and 28,617 edges). Focusing on genes in this network, we extract from BioMart 2,219 associations between 1,441 diseases and 1,271 genes. Directly applying the Bayesian regression approach to this high confident network yields mean rank ratios 0.1373 and 0.1380 (AUC scores 0.8717 and 0.8694) for random controls and linkage intervals, respectively. Though these results are slightly better than those of the Bayesian integration method, the coverage of this high confident network is apparently much lower than that of the Bayesian integration approach. From these results, we conclude that the Bayesian regression approach is effective in integrating multiple PPI networks for prioritizing disease genes.

It is of interest to see how much individual PPI networks contribute to the integration approach. For this purpose, we repeat the cross-validation experiments by integrating every four data sources and see how the exclusion of the remaining network affects the prioritization results. We find that all data sources have positive contributions to the integration approach, because mean rank ratios increase and AUC scores drop when any of the five data source is excluded. Results also suggest the order of the data sources according to their contributions (differences in cross-validation results) as follows: HPRD > BIND > BioGRID > IntAct > MINT. It is not surprising that HPRD contributes most to the integration method, because HPRD has the largest coverage and highest performance in previous validations. It is also not surprising that MINT has the least contribution, and IntAct has the second least contribution, because both coverage and performance of these two data sources are not high. However, it is not obvious that BIND contributes more than BioGRID, because individually, BioGRID has higher coverage and performance than BIND. To understand this observation, we analyze the relation between the five data sources and find that Bayes factors calculated from HPRD and BioGRID are highly correlated (Pearson's correlation coefficient = 0.9770). Therefore, the removal of BioGRID, when HPRD is presented, will not significantly affect the performance of the integration method.

Finally, we study whether the integration approach is biased toward well characterized genes, that is, genes appearing in more data sources tend to have higher ranks. We group all genes in a validation procedure into 10 categories according their ranks such that the *i*-th category contains genes ranked at ((*i* - 1)×10%,*i*×10%]. For each category, we group genes according to the number of PPI networks containing the genes such that the *j*-th category contains genes appearing in exact *j* networks. We then perform pair-wise Pearson's chi-squared tests against the alternative hypothesis that frequencies of genes appearing in different number of PPI networks are different across different rank categories. Results show that for the cross-validation experiment on linkage intervals, the minimum *p*-value produced by the series of chi-square tests is 0.0909, suggesting that we cannot reject the null hypothesis. Similar results are obtained for the cross-validation experiment on random controls. We therefore conclude that the integration approach is not biased toward well characterized genes. In other words, genes appearing in more data sources do not tend to have higher ranks.

## Conclusions and discussion

In this paper, we propose a Bayesian regression approach that relies on the linear relationship between disease similarity and gene proximity to prioritize candidate genes. We show that gene proximity, as the diffusion distance obtained from some PPI network, implies disease similarity, and we perform a series of leave-one-out cross-validation experiments to demonstrate the effectiveness of the proposed approach. We show that the Bayesian regression approach can achieve much higher performance that an existing CIPHER approach and the ordinary regression method. We also use the proposed approach to integrate multiple PPI networks, to achieve higher coverage while maintaining superior performance. Our contribution in this paper therefore lies in the following points: (1) systematic validation of the assumption that gene proximity in a PPI network implies disease similarity; (2) the Bayesian regression approach that greatly improves the performance in prioritizing disease genes, in comparison with a previous CIPHER approach; (3) detailed analysis of the effectiveness of five widely-used PPI networks in prioritizing disease genes; (4) a simple yet effective method to integrate multiple PPI networks into a single prioritization model.

Certainly, our approach can be further studied from the following aspects. First, the main reason of using conjugate priors in the Bayesian regression model is to seed for analytic solutions and thus alleviate the computational burden in the calculation of Bayesian factors. Although this formulation shows great success, it is known that the specification of prior is intrinsically complicated and subjective. The main consideration here is that the posterior mean and variance should not depend on the units in which the disease similarities are measured and should also be invariant to the shift of the response variable. Therefore, one can consider the use of Jeffreys prior instead of the conjugate prior. By doing this, a Markov chain Monte Carlo (MCMC) approach would be necessary in the calculation of the marginal likelihood, and thus the computational burden could be high.

Second, it is conceptually straightforward to extend the Bayesian regression model to infer interactive effects of multiple genes on a query complex disease. For example, we can enumerate pair-wise combinations of all candidate genes and calculate a Bayes factor for each combination to infer the interactive effects of two genes on a query disease. Nevertheless, the challenge will come from the computational feasibility, because the number of combinations of even a small number of candidate genes will be large.

Third, the means of dealing with missing data (setting to zeros) in the proposed approach, though simple, is kind of naïve. When more data sources are integrated, the overlap of genes between data sources will typically be lower, and thus a more effective method for dealing with missing data is desired. One possible solution is to interpolate missing data use the mean or median of observed data. Another possible solution is to rank candidate genes using data sources individually and then aggregate the ranks, as what is done in existing literature [6]. Both methods have their own advantages and disadvantages, and a comprehensive comparison study is necessary in order to obtain detailed understanding of these possible solutions.

Finally, there have been a lot of large scale data produced by high-throughput techniques. To mention a few, sequences of most human protein-coding genes are known; mapping of human genes to Gene Ontology (GO) is available; expression profile for most human genes across various conditions has been obtained. How to extend the proposed approach to integrate these data sources will be one of the directions in our future work.

## Competing interests

No conflicts of interests declared.

## Authors' contributions

WZ implemented the method, collected the results, and wrote the manuscript. FS designed the research and wrote the manuscript. RJ designed the research and wrote the manuscript. All authors read and approved the final manuscript.
